# When “Everything Is Perfect”: A Family Perspective on Communication, Expectations, and Unplanned Cesarean Delivery

**DOI:** 10.7759/cureus.117779

**Published:** 2026-10-11

**Authors:** Jordan Scott, Steven Powers

**Affiliations:** 1 Osteopathy, Liberty University College of Osteopathic Medicine, Lynchburg, USA; 2 Obstetrics and Gynecology, Chesapeake Regional Medical Center, Chesapeake, USA

**Keywords:** cesarean delivery, childbirth, patient-centered communication, patient experience, shared decision-making

## Abstract

The unpredictability of labor presents challenges for patients, families, and clinicians. While obstetric providers cannot predict individual delivery outcomes with certainty, communication can help patients develop realistic expectations and prepare for multiple possible birth experiences. We describe our family’s experience with an induction of labor followed by an unexpected transition to cesarean delivery. Although maternal and neonatal outcomes were favorable, we felt unprepared for the possibility of operative delivery until prolonged labor and pushing made cesarean delivery a realistic consideration. Reflecting on this experience, and subsequently through the perspective of medical training, highlighted for us the distinction between reassurance and preparation. Drawing on our family's experience and the literature on informed consent and shared decision-making, this article reflects on the potential value of communicating uncertainty while preserving optimism, without assuming that this approach necessarily improves patient preparedness.

## Editorial

Childbirth is both one of life's most anticipated milestones and one of its most unpredictable experiences. While modern obstetric care has greatly improved maternal and neonatal outcomes, no clinician can predict with certainty how an individual labor will unfold [1]. As expectant parents, we recognized that uncertainty is an inherent part of childbirth. What we did not anticipate was how profoundly our experience would be shaped not by the unexpected outcome itself, but by how unprepared we felt for the possibility of that outcome.

Our daughter was ultimately born safely by cesarean delivery after a prolonged induction and failed progression of labor. We remain profoundly grateful for that outcome. However, in the weeks and months that followed, we realized that what lingered most was not disappointment about the mode of delivery, but our retrospective perception that we had not adequately understood cesarean delivery as a realistic possibility. Looking back, we did not wish for certainty. We wished for a better understanding of uncertainty.

This article presents our family's experience from my perspective as a husband. My subsequent obstetrics and gynecology rotation as a medical student provided an opportunity to reflect on that experience within the broader context of patient-centered communication and shared decision-making. It is not intended to suggest that cesarean delivery could or should have been predicted, but rather to explore how communication surrounding uncertainty shaped our experience and prompted us to consider how similar conversations might help other families prepare for the possible paths childbirth can take.

Our experience

My wife entered pregnancy healthy, active, and excited to become a first-time mother. As a former Division I athlete, she approached pregnancy with careful preparation and determination. Throughout pregnancy, we attended routine prenatal visits and looked forward to the vaginal birth we had carefully planned.

As the third trimester progressed, several new challenges emerged. My wife developed significant pubic symphysis pain that increasingly affected her daily activities. She later developed pruritic urticarial papules and plaques of pregnancy (PUPPP), followed by a diagnosis of intrahepatic cholestasis of pregnancy requiring induction of labor at 38 weeks and 3 days. A late ultrasound demonstrated a fetal head circumference measuring approximately the 98th percentile. During labor, we also learned that polyhydramnios had been documented. While each finding was discussed individually, we did not understand whether or how these findings might influence the course of labor or the possibility of cesarean delivery. We recognize that these findings alone do not establish that cesarean delivery should have been anticipated.

Despite the need for induction, we remained optimistic. My wife had spent months preparing physically and emotionally for a vaginal birth and remained committed to that goal throughout labor. She labored for approximately 18 hours before pushing continuously for nearly four hours. Watching her continue pushing despite increasing exhaustion was emotionally difficult, particularly as it became increasingly clear that labor was not progressing as we had hoped.

Throughout prenatal care and the early stages of labor, we recall discussions focused primarily on the planned vaginal delivery and the clinical indication for induction. We do not recall a detailed discussion of cesarean delivery as a possible alternative or contingency plan before prolonged pushing. However, this account reflects our recollection and understanding of those conversations rather than an independent review of the medical record.

Only after prolonged pushing do we recall having our first substantive conversation about cesarean delivery. We were informed that, despite my wife's extraordinary effort, the baby's head was not descending adequately and that successful vaginal delivery was unlikely. Although earlier counseling may have occurred, we had not understood cesarean delivery to be a realistic possibility until that moment.

The transition from preparing for a natural birth to preparing for surgery occurred within a matter of minutes. We ultimately agreed to proceed with cesarean delivery, and our daughter was delivered safely, weighing 8 pounds 3 ounces. We remain profoundly grateful for the care that resulted in a healthy mother and baby. Although the clinical outcome was positive, what remained with us after delivery was not disappointment about the mode of birth itself. Rather, it was the feeling that we had not been prepared for the possibility of that outcome until it became an urgent reality.

Reflection

In the months following our daughter's birth, we reflected on our experience often. Our frustration was never rooted in the need for a cesarean delivery. We recognize that labor is inherently unpredictable and that no clinician can determine with certainty which patients will ultimately require operative delivery [1]. Looking back, we remain grateful for the clinical care that resulted in a healthy mother and baby.

Instead, our reflections centered on how we experienced the uncertainty surrounding labor. Throughout pregnancy, we interpreted repeated reassurance that everything was progressing well as reassurance that the birth we had planned remained the most likely outcome. When we first understood cesarean delivery to be a realistic possibility after prolonged labor and pushing, we found ourselves making an important decision while physically exhausted and emotionally overwhelmed. In retrospect, we realized that we had spent months preparing for one possible birth experience rather than preparing for the range of outcomes that childbirth can involve [2].

Several months later, I completed an obstetrics and gynecology rotation as a medical student. Caring for patients from the clinical side of the bedside provided an opportunity to revisit our own experience from a different perspective. I encountered patients whose questions echoed many of our own-not necessarily why a cesarean delivery had become necessary, but why they had felt so unprepared for that possibility. Although every pregnancy and labor is unique, those conversations reinforced my appreciation for the importance of communication throughout pregnancy and labor [3].

Our experience ultimately changed how we think about patient-centered communication. We no longer view reassurance and preparation as competing goals. For our family, certainty about how labor would unfold was neither possible nor what we needed. We would have valued honest communication about the range of possible outcomes while preserving hope for the birth we envisioned. That balance between reassurance and preparation might have made our experience less emotionally overwhelming, although we cannot establish whether earlier discussions would have changed our experience or the clinical decisions ultimately made [4].

Although our family's experience represents a single perspective, it prompted us to reflect on several communication practices that may be worth considering for patients and families facing similarly unpredictable labor courses [5].

First, reassurance and preparation need not be opposing goals. Throughout pregnancy, we found comfort in being reassured that everything was progressing appropriately. In retrospect, however, reassurance alone did not prepare us for the possibility that labor might ultimately require a different path than we had envisioned. Our family's experience led us to reflect on the value of discussing uncertainty while maintaining optimism. Although informed consent and shared decision-making are supported by professional guidance [4], our experience cannot establish whether this particular communication approach improves patients' preparedness.

Second, the timing and extent of discussions about potential labor outcomes warrant consideration in light of individual clinical circumstances, patient preferences, and the likelihood of operative delivery. Introducing cesarean delivery as one possible outcome during prenatal care or early labor may be appropriate in some circumstances and could provide an opportunity to discuss expectations before decisions become urgent. Our experience raises this as a patient-centered communication consideration rather than a recommendation that all patients receive identical counseling at a particular stage of pregnancy or labor [2,3].

Finally, our experience reinforced that patient-centered communication extends beyond explaining medical decisions. It also involves helping patients understand how uncertainty influences clinical decision-making and preparing them for the range of outcomes that may occur despite everyone's best efforts. Our experience led us to consider whether thoughtful communication might influence how some patients and families experience that journey, even when the clinical course itself is unchanged.

We recognize that every pregnancy and labor is unique and that no single communication strategy will meet every patient's needs. This reflection is limited to one family's retrospective experience, supplemented by the author's subsequent clinical training. It cannot establish the prevalence, causes, or effects of inadequate communication in obstetric care or demonstrate that earlier discussion of cesarean delivery would improve patient experiences. Additionally, our account relies on retrospective recollection and does not independently establish the content or timing of all communication provided by the clinical team. Our reflections should therefore be understood as considerations for further discussion rather than evidence of a particular communication strategy's effectiveness.

Our family's experience reinforced a lesson that has remained with us long after our daughter's birth: while the outcome of labor cannot always be predicted, we wish we had been better prepared for the uncertainty that accompanied it. Earlier conversations about the range of possible labor outcomes would not necessarily have changed our daughter's delivery or the clinical decisions that were ultimately made. However, we believe they might have changed how we experienced that journey, although this potential benefit remains hypothetical and cannot be established from our experience [4].

We hope our experience encourages continued reflection on how communication, expectation-setting, and shared decision-making shape the patient experience during childbirth. For our family, acknowledging uncertainty earlier would not have diminished hope for the birth we envisioned. We hope our experience encourages consideration of how individualized communication might help patients and families feel informed and supported while recognizing that their needs, preferences, and clinical circumstances vary.
